# Idiopathic Facial Aseptic Granuloma: A Case Report

**DOI:** 10.7759/cureus.95862

**Published:** 2025-10-31

**Authors:** João Patrocínio, Pedro Miguel Garrido, Sara Turpin, Óscar Dias, Sónia Fernandes

**Affiliations:** 1 Department of Dermatology, Unidade Local de Saúde de Santa Maria, Lisbon, PRT; 2 Department of Pathology, Hospital SAMS, Lisbon, PRT; 3 Otorhinolaryngology Clinic, Hospital SAMS, Lisbon, PRT

**Keywords:** benign dermatosis, childhood rosacea, facial granuloma, granulomatous inflammatory infiltrate, pediatric dermatosis

## Abstract

Idiopathic facial aseptic granuloma (IFAG), an exclusive pediatric dermatosis, is typically characterized by one or more asymptomatic erythematous nodules on the face. While its etiology remains elusive, there are suggestions that it could be a manifestation of childhood rosacea. This case report sheds light on a five-year-old male with an erythematous nodule on the right nasolabial fold, conclusively diagnosed as IFAG through histopathological examination. A detailed overview of the clinical approach, treatment, and outcome is presented, contributing valuable insights to the sparse literature on this rare condition.

## Introduction

Idiopathic facial aseptic granuloma (IFAG) is a distinctive and rare dermatological entity primarily observed in the pediatric age group, typically presenting as one or more asymptomatic, erythematous nodules on the face [1-6]. Despite extensive research efforts, the exact etiology and pathogenesis of IFAG remain largely unknown [2]. However, an increasing body of evidence suggests that IFAG might represent a unique manifestation within the spectrum of childhood rosacea, given associated clinical features like chalazion and eyelid involvement [1,5,7,8]. Historically, IFAG was sometimes referred to as *pyodermite froide du visage* due to its cold abscess-like appearance, a term reflecting its mild inflammatory nature [6,9]. Given its unique characteristics, rarity, and the ongoing debate surrounding its precise nature and origins, IFAG continues to be a significant subject of interest for clinicians and researchers dealing with pediatric skin disorders. Regarding treatment, there is limited documentation on management strategies for refractory cases unresponsive to antibiotics. This case report aims to further contribute to the understanding of this condition by detailing the role of histopathological examination in confirming its diagnosis and demonstrating the efficacy of surgical intervention in the successful management of a refractory case, thereby providing valuable insights into the clinical implications of IFAG.

## Case presentation

**Figure 1:**
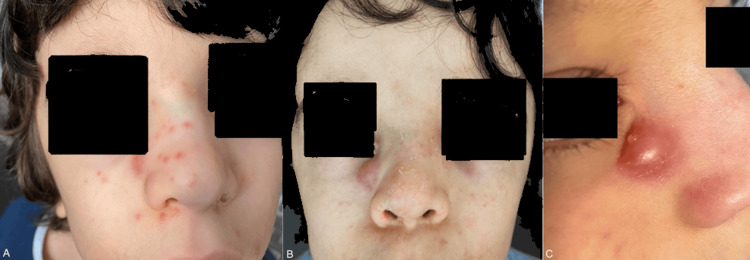
Clinical image. (A) Initial clinical presentation: erythematous nodule of elastic consistency on the right nasolabial fold, with 10 mm of greatest axis, associated with erythematous papules in the surrounding skin; (B and C) Clinical presentation after six months: increase in the volume of the nodule and decrease in the number of peripheral papules and erythema (B); detail of the erythematous nodule, with suppuration (C).

The patient was a five-year-old male, brought to our clinic due to an erythematous nodule on the right nasogenian region, evolving for six months (Figure 1). The nodule was asymptomatic, with no associated pain or pruritus, although there were reported episodes of suppuration. He had been previously treated with local antibiotics, including metronidazole gel (7.5 mg/g), and systemic antibiotics, comprising amoxicillin+clavulanic acid and clarithromycin, without noticeable improvement. The child's medical history was significant for prematurity and extremely low birth weight.

**Figure 2:**
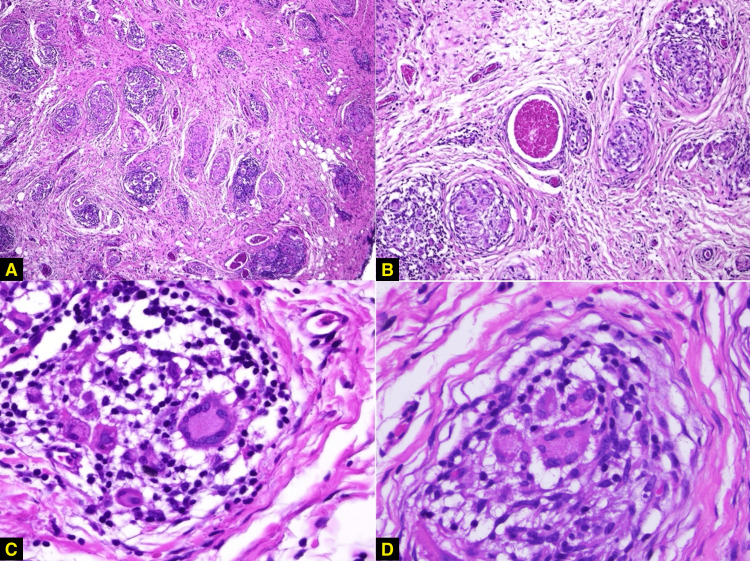
Histopathological examination. (A, B) Chronic granulomatous inflammatory infiltrate in the mid and deep dermis, with abundant epithelioid granulomas and no evidence of necrosis. The absence of necrosis distinguishes idiopathic facial aseptic granuloma (IFAG) from infectious granulomas (H&E, ×40; H&E, ×100). (C, D) Presence of Langhans-type and foreign body-type giant cells (H&E, ×400).

Upon physical examination, a well-demarcated erythematous nodule with elastic consistency was found in the right nasolabial fold, measuring approximately 10 mm in its longer axis. It was surrounded by a few erythematous papules. The decision was made to perform an excision of the lesion. Histopathological examination revealed a chronic granulomatous inflammatory infiltrate in the mid and deep dermis (Figure 2). There was an abundance of epithelioid granulomas, with no evidence of necrosis, and the presence of both foreign body-type giant cells and Langhans-type cells. These findings corroborated the clinical suspicion of IFAG.

**Figure 3:**
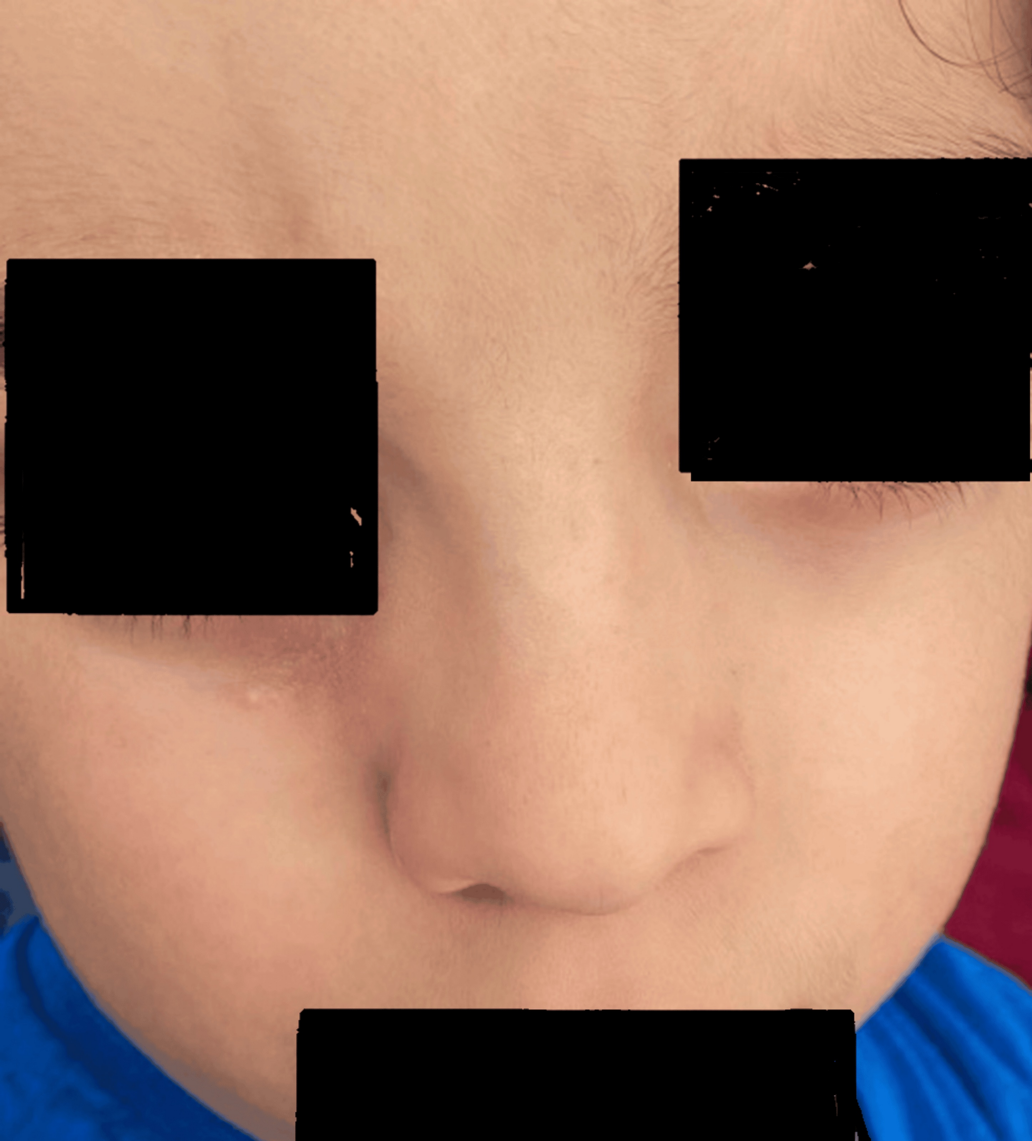
Six-month follow-up. An excellent cosmetic and functional outcome was achieved following surgery. Complete resolution of the dermatosis was observed, with no evidence of recurrence after six months of follow-up.

Post-surgery, as part of the ongoing treatment strategy, ivermectin cream was prescribed for application on the residual satellite papules. After a diligent six-month follow-up period, there was complete resolution of the main nodule due to surgical excision, and the satellite papules also resolved with ivermectin treatment, resulting in overall dermatosis resolution. The surgical scar healed excellently, achieving an aesthetically pleasing outcome (Figure 3).

## Discussion

IFAG remains a rare dermatosis predominantly affecting the pediatric population, with a limited number of cases documented in medical literature [1,4]. The diagnostic journey for IFAG is often challenging due to its varied clinical presentations and overlap with numerous other conditions. As observed in our case, where a five-year-old male presented with an erythematous nodule, distinguishing IFAG from other facial nodules in children is critical [4]. Differential diagnoses commonly considered include localized pyodermas, benign tumors such as pilomatricoma, dermoid or epidermoid cysts, nodulocystic acne, vascular malformations, pyogenic granulomas, Spitz nevi, xanthogranulomas, and even infectious etiologies like bacterial (e.g., *Leishmania* or mycobacteria) or fungal infections, especially in immunocompromised patients [3,4,9]. While these conditions can have similar appearances, their clinical courses and required treatments differ significantly, necessitating accurate differentiation to prevent negative evolutions when not promptly diagnosed and treated [3]. The histopathological examination, revealing a chronic granulomatous inflammatory infiltrate with epithelioid granulomas and giant cells in the mid and deep dermis, was pivotal in our case for confirming the clinical suspicion of IFAG, aligning with the diagnostic approach highlighted in the literature [3,9].

The clinical course of IFAG is generally benign and chronic, with a notable tendency for spontaneous resolution, often within a year, as reported in various studies [1-3]. This natural history often leads to a conservative approach, where biopsy and surgical excision are frequently deferred, particularly given the cosmetically sensitive facial location [1]. However, our case, which involved a nodule evolving for six months and unresponsive to prior local and systemic antibiotic treatments, represents a refractory presentation. In such instances, while no universally *well-defined treatment* has emerged, the literature supports a range of therapeutic strategies [2,3]. Conservative approaches are generally preferred to avoid aggressive therapies [2,3]. Beyond the initial, unsuccessful antibiotic regimen in our patient, other documented treatments include various topical and systemic antibiotics, as well as isotretinoin [6]. For example, one report detailed a case of IFAG that failed oral doxycycline but responded to a combination of oral 13-cis-retinoic acid, oral trimethoprim-sulfamethoxazole, and intralesional triamcinolone injections [6]. Our case further demonstrates that surgical intervention, leading to complete resolution and an excellent aesthetic outcome, plays a crucial role in the management of chronic and refractory cases, a finding consistent with studies emphasizing the importance of surgery in such situations [10]. While quantitative data specifically reporting the curative rates of surgical outcomes for IFAG in the literature are limited, successful resolution following excision, particularly in resistant cases, is a recognized outcome.

The choice of ivermectin cream for application on satellite papules in our case was largely guided by the increasing body of evidence suggesting IFAG's potential inclusion within the spectrum of childhood rosacea [1,5,7,11]. Ivermectin is known for its anti-inflammatory and acaricidal properties, particularly against Demodex folliculorum mites, which are implicated in the pathogenesis of rosacea [12,13]. Therefore, its use in IFAG, when a linkage to rosacea is suspected or observed, represents a targeted therapeutic approach based on pathophysiological hypotheses rather than solely empirical treatment.

Regarding the long-term prognosis, the current follow-up of six months in our case demonstrated complete resolution and an excellent aesthetic outcome. While this provides promising immediate results, longer follow-up data would undeniably strengthen the claims of complete resolution and sustained lack of recurrence, as the natural history of IFAG can extend over several months to a year. Studies focusing on long-term outcomes for IFAG patients exist, though specific recurrence rates post-surgical intervention are not extensively quantified in the literature [10].

The precise etiopathogenesis of IFAG remains unclear [1,7]. However, several hypotheses contribute to our understanding. The strong association with childhood rosacea is one prominent theory, supported by similar clinical features and histopathological findings [5]. Histologically, IFAG presents with a chronic granulomatous inflammatory infiltrate, characterized by perifolliculitis, granulomas, and follicular involvement, aligning with features observed in granulomatous rosacea [5]. This suggests an inflammatory process centered around hair follicles. Beyond the rosacea linkage, other proposed pathophysiologic mechanisms include IFAG as a granulomatous process arising around an embryological remnant [14], or reactive inflammation following trauma. While specific hypotheses directly addressing *immune dysregulation* are not explicitly defined in the literature for IFAG, the consistent histopathological finding of chronic granulomatous inflammation inherently points to a complex immune response and dysregulation as part of its underlying pathology.

## Conclusions

This case report on IFAG underscores the clinical manifestations, challenging diagnosis, and effective management strategies for this unusual pediatric dermatosis. Our detailed account of a five-year-old male, diagnosed through crucial histopathological examination after presenting with a persistent facial nodule, contributes to the limited literature on this condition by illustrating the diagnostic complexities and the varied differential diagnoses that clinicians must consider. Furthermore, this study highlights the beneficial role of surgical intervention, which, in our refractory case, led to complete resolution and an excellent cosmetic outcome where prior conservative treatments had failed. This outcome emphasizes that while many cases may resolve spontaneously, when IFAG is refractory to antibiotics, surgical excision can be both diagnostic and curative. Ultimately, this report serves to encourage clinicians, particularly those in pediatric dermatology, to maintain a high index of suspicion for IFAG, enabling prompt diagnosis and the implementation of appropriate, individualized management strategies to ensure optimal patient outcomes. Future research should focus on fully elucidating the underlying immunopathogenesis of this intriguing condition and exploring non-invasive diagnostic methods.
